# Supplementary material for: Generalization of neoantigen-based tumor vaccine by delivering peptide-MHC complex via oncolytic virus
Source: EMBO Mol Med. 2025 Apr 7;17(5):1118–52. doi: 10.1038/s44321-025-00225-3 (PMC12081622; doi:10.1038/s44321-025-00225-3)
Supplement: Supplementary file 1 — Appendix [file 44321_2025_225_MOESM1_ESM.pdf]

Appendix Data

Table of contents:

APPENDIX FIGURES	PAGE
Appendix Fig. S1	1
Appendix Fig. S2	2
Appendix Fig. S3	3
Appendix Fig. S4	4
Appendix Fig. S5	5
Appendix Fig. S6	6
Appendix Fig. S7	7
Appendix Fig. S8	8
Appendix Fig. S9	9
Appendix Fig. S10	10
Appendix Fig. S11	11-12

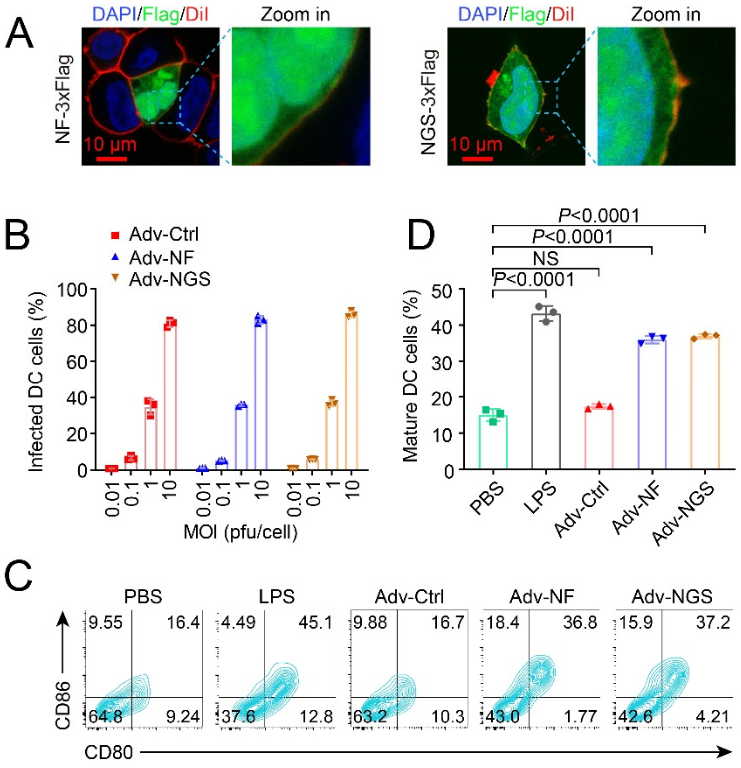

Appendix Fig. S1 Validation of adenovirus-vectored neoantigen vaccines.

(A) Immunofluorescence staining showed the overexpression and cellular distribution of NF-3×Flag and NGS-3×Flag in HEK293T cells after 24 h of plasmid transfection. Blue signal indicated DAPI staining of the cellular nucleus; Green signal indicated immunofluorescence staining of the Flag tags; Red signal indicated DiI staining of the cellular membrane. Scale bar: 10 μm. (B) Quantification of the percentage of mCherry-positive DCs via flow cytometry after 48 h exposure to different MOIs of indicated adenoviruses (n=3). (C) Quantification of DC maturation via flow cytometry after 72 h exposure to 2 MOI of indicated adenoviruses. Treatment with PBS and LPS (1 μg/ml) were respectively referred to as the negative and positive control (n=3). (D) Statistical analysis of the results in Figure C. Statistical analysis was performed using one-way ANOVA (D). Data are shown as mean±SD for 3 biological replicates.

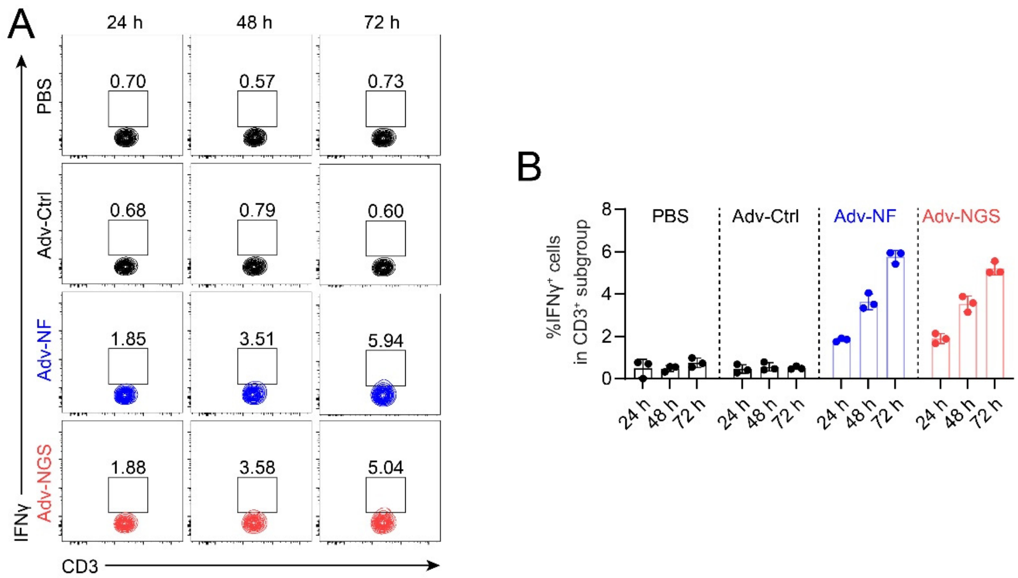

Appendix Fig. S2 Flow cytometry analysis demonstrated T cell responses during the DC-mediated antigen presentation.

(A) Flow cytometry analysis demonstrated T cell reactivity toward the antigen presentation by pre-treated DCs. Treated with PBS or 2 MOI indicated adenoviruses for 72 h, 1×10^4 DCs were co-cultured with 5×10^4 freshly prepared SMNCs for in vitro antigen presentation. The cells were collected at 24 h, 48 h, or 72 post the co-culture and subjected to flow cytometry analysis. (B) Numeric results of Figure A (n=3). Data are shown as mean±SD for 3 biological replicates.

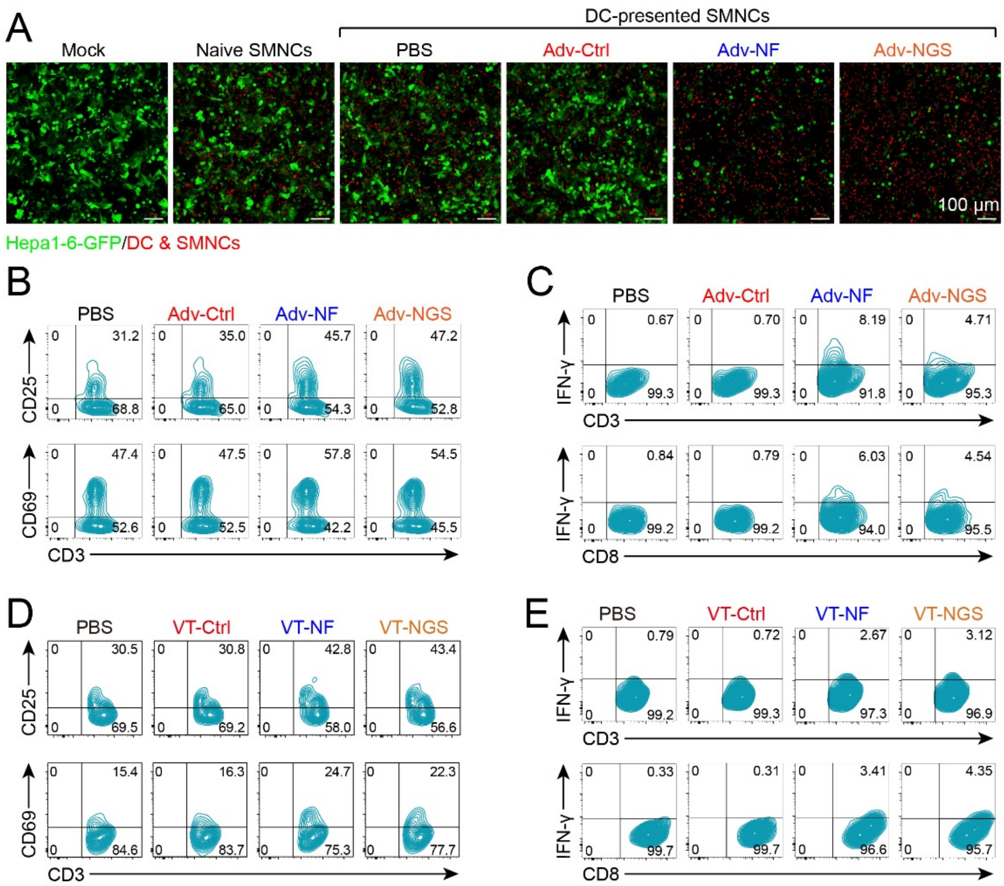

Appendix Fig. S3 Fluorescent images and flow cytometry analysis characterized the generation of functional T cells.

(**A**) Fluorescent images visualized the elimination of Hepa1-6 cells by antigen-presented SMNCs. Green signals indicated Hepa1-6 stably expressing GFP (Hepa1-6-GFP), while red signals indicated DCs and SMNCs stained with CM-Dil (1 μM, 30 min at room temperature). Scale bars, 100 μm. Mock indicated control group containing only Hepa1-6-GFP cells, Naïve SMNCs indicated Hepa1-6-GFP cells co-cultured with naive DCs and SMNCs. (**B** and **C**) Flow cytometry analysis demonstrated the percentage of activated T cells after in vitro antigen presentation and the co-culture with Hepa1-6 cells (n=3). (**D** and **E**) Flow cytometry analysis demonstrated the percentage of activated vaccine-primed T cells after the co-culture with Hepa1-6 cells (n=3).

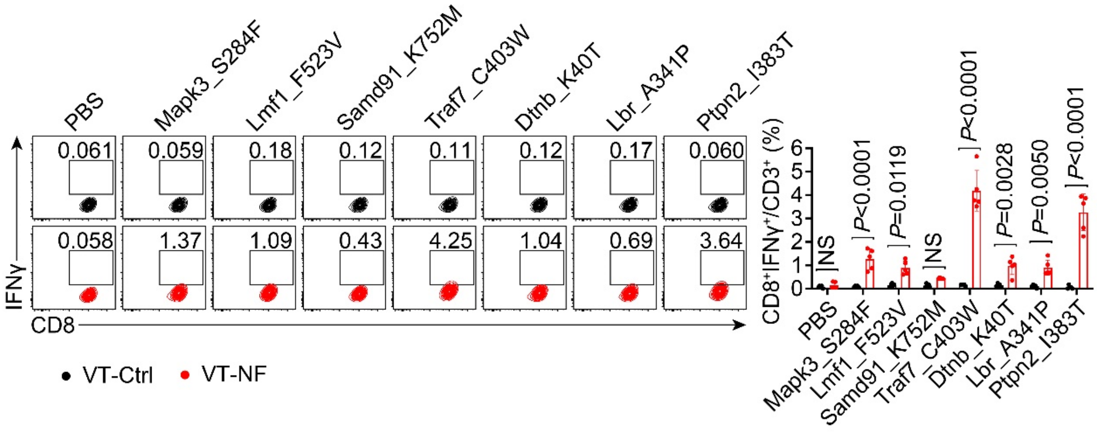

Appendix Fig. S4 Immunogenicity of neoepitopes within adenoviral vaccine.

DCs were pulsed with 100 nM of indicated neoantigen peptides for 24 h before subjecting to the co-culture with VT-Ctrl or VT-NF cells at a ratio of 1:5 for additional 24 h. The cell culture medium was supplemented with GolgiStop Protein Transport Inhibitor (4 μl per 6 ml culture medium) 4 h before the collection of the cells. Subsequent to the collection, the cells were stained with fluorophore-conjugated antibodies for flow cytometry analysis (n=5). Statistical analysis was performed using two-way ANOVA. Data are shown as mean±SD.

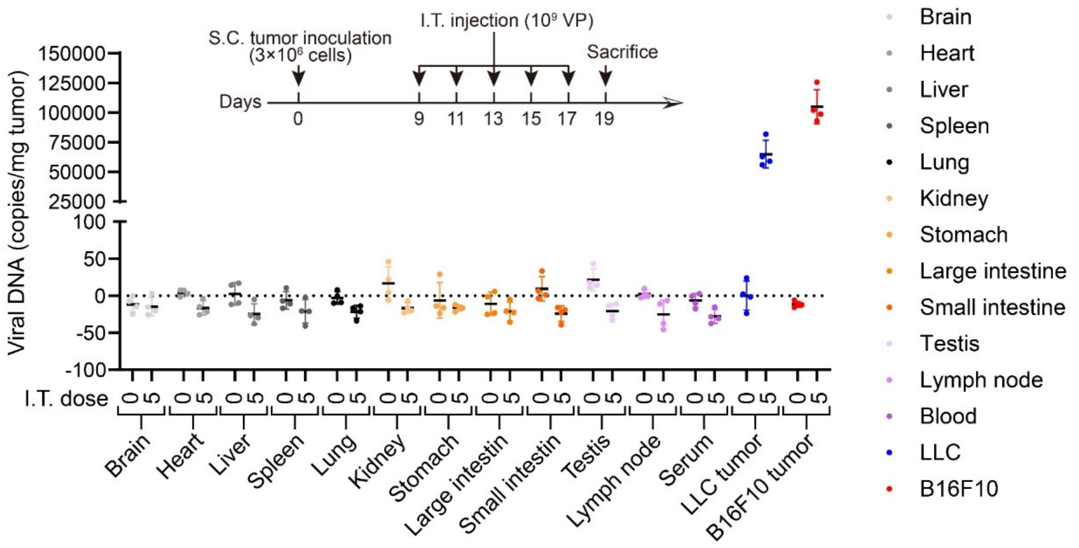

Appendix Fig. S5 Tissue distribution of intratumorally injected adenovirus.
PBS and Adv-Ctrl (10^9 VP) were injected into LLC- or B16F10-established tumors five times before the mice were sacrificed. Genomic DNA was extracted from the tumors and primary organs of the mice, and a qPCR assay was performed to quantify the amount of viral DNA. Treatment with PBS and Adv-Ctrl is referred to as I.T. dose 0 and I.T. dose 5, respectively (n=4). Data are shown as mean±SD.

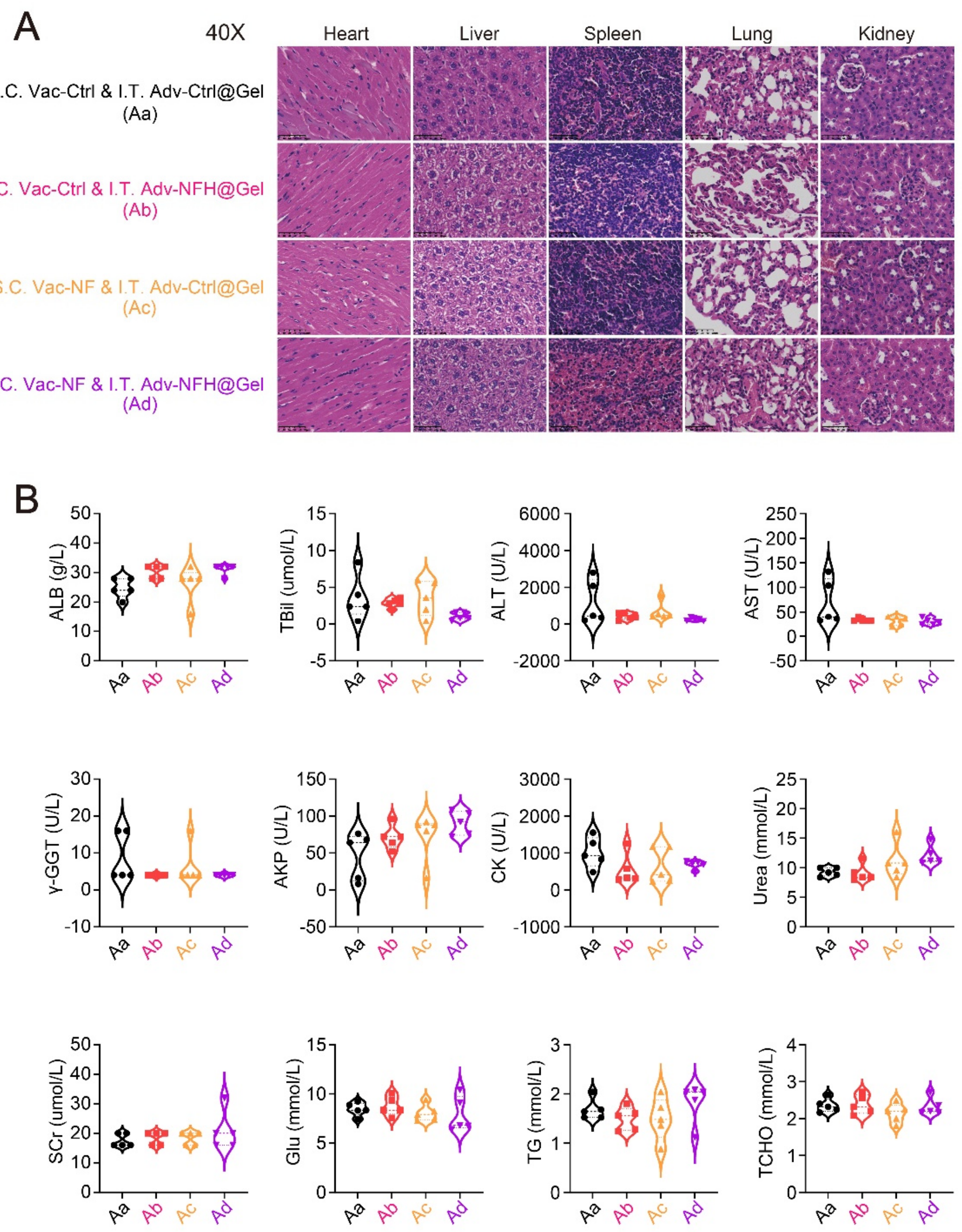

Appendix Fig. S6 Safety profile of the integrative therapy in C57BL/6 mice.
(A) HE staining of mouse organs after the integrative therapy. Scale bar: 50 μm. (B) Serum biochemical analysis after the integrative therapy (n=5). ALB, albumin; TBil, total bilirubin; ALT, alanine amiotransferase; AST, aspartate aminotransferase; γ-GGT, Gamma-glutamyl transferase; AKP, Alkaline phosphatase; CK, creatine kinase; SCr, Serum creatinine; Glu, Glucose; TG, triglyceride; TCHO, total cholesterol. Statistical analysis was performed using one-way ANOVA without observing significant difference. Data are shown as violin plot for 5 biological replicates.

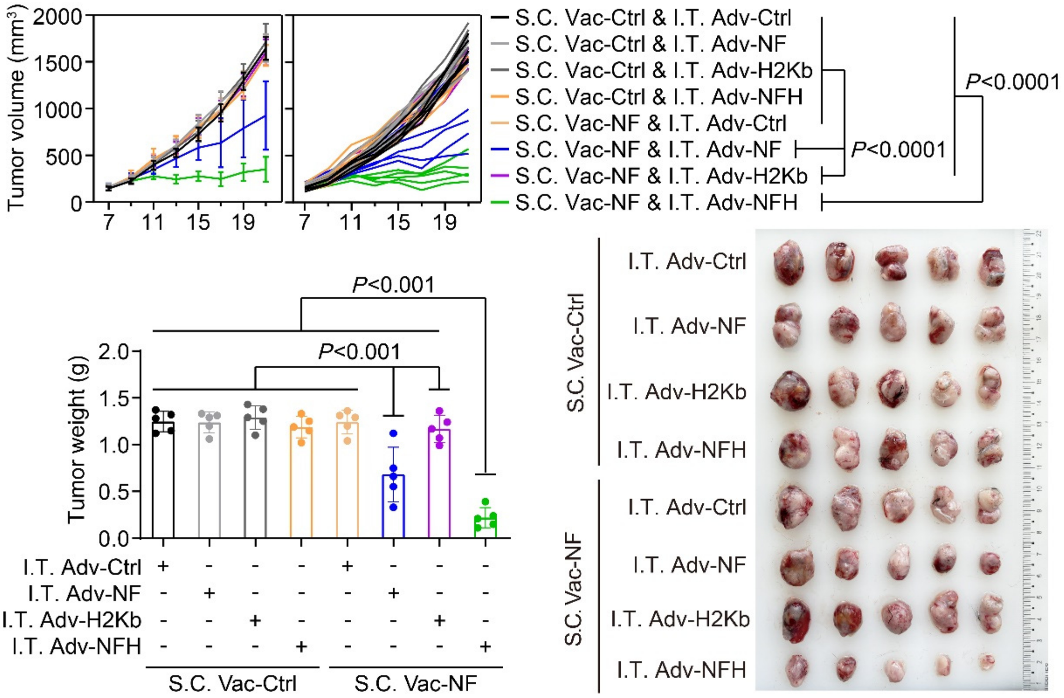

Appendix Fig. S7 Integrative immunotherapy in LLC-established tumor model. The experiments were performed by following the procedure as described in the Fig. 4C (n=5 mice per group). Statistical analysis was performed using two-way ANOVA (tumor growth curve) or one-way ANOVA (tumor weight). Data are shown as mean±SD.

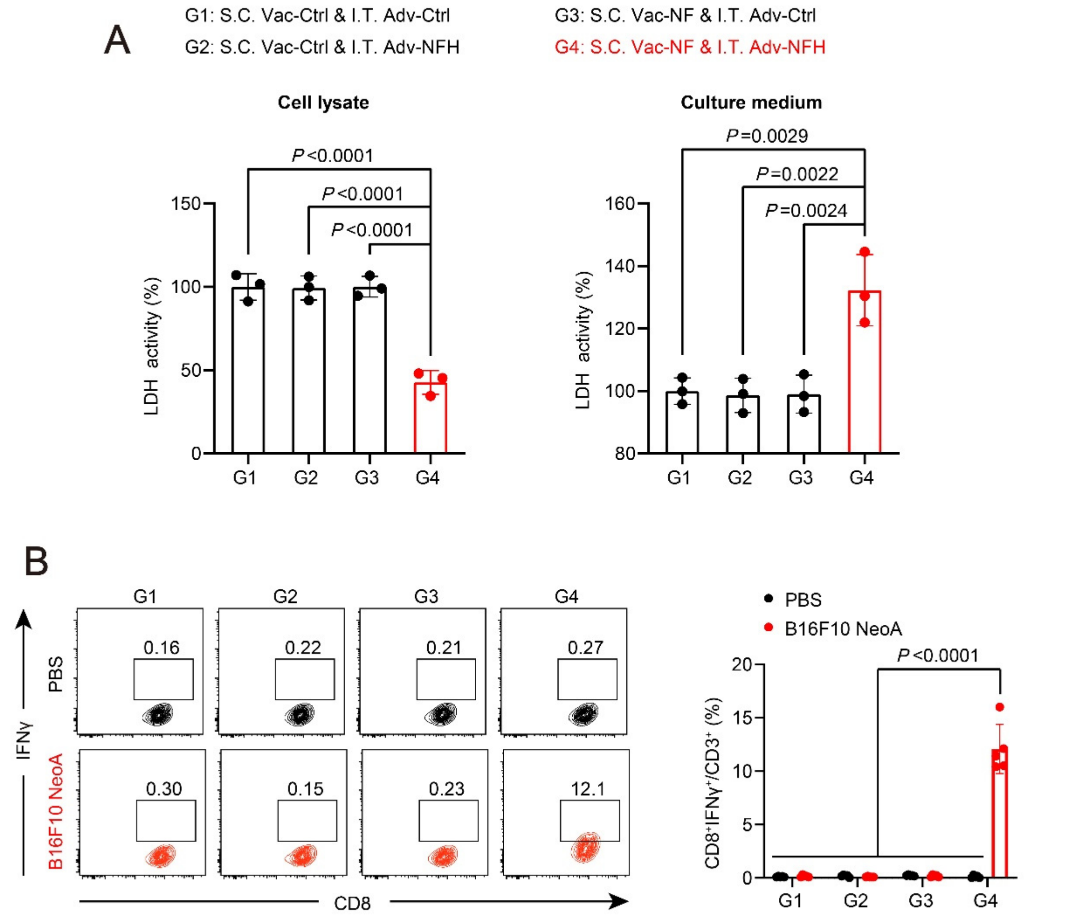

Appendix Fig. S8 T cells obtained from mice treated by integrative immunotherapy exhibited B16F10-specific cytotoxicity.

(**A**) B16F10 cells were subcutaneously inoculated into the right flanks of C57BL/6 mice, which were pre-immunized with one dose of Vac-Ctrl or Vac-NF (2×10^9 VP per mouse) for 14 d. Adv-Ctrl or Adv-NFH (10^9 VP/dose) were intratumorally injected into the established tumor tissues on day 9, day 11, day 13, day 15 and day 17 post tumor inoculation, respectively. SMNCs were obtained from the treated mice on day 20, stimulated with anti-CD3ε/anti-CD28 antibodies and IL-2 for 3 d, and maintained in KPM581 containing 1000 U/ml IL-2. Thereafter, the obtained T cells were co-cultured with B16F10 (10^5 cells) at a ratio of 3:1 in a 24-well plate for 48 h before subjecting to LDH cell cytotoxicity assay (n=3). (**B**) 6 neoantigen peptides of B16F10 cells were selected based on their predicted MHC-I IC50 (14.98 nM to 72.41 nM), including Pcmtd1_P222L (VSFAPLVQL), Haus6_L176V (VARNRFVQI), Ncor1_H673P (FNYKRRPNL), Vps13c_S1256P (SSLPTNAVVV), Ctsd_G397S (VSFANAVVL), and Nsun2_K72M (KILRMSPL). Thereafter, DCs obtained from naïve mice were treated a pool of neoantigen peptides (B16F10 NeoA, 10 nM/peptide) for 24 h, and co-cultured with the freshly prepared SMNCs of treated mice at a ratio of 1:5 for additional 24 h. Subsequently, the cells were stained with fluorophore-conjugated antibodies, and subjected to flow cytometry analysis (n=5). Statistical analysis was performed using one-way ANOVA (A) or two-way ANOVA (B). Data are shown as mean±SD for 3-5 biological replicates.

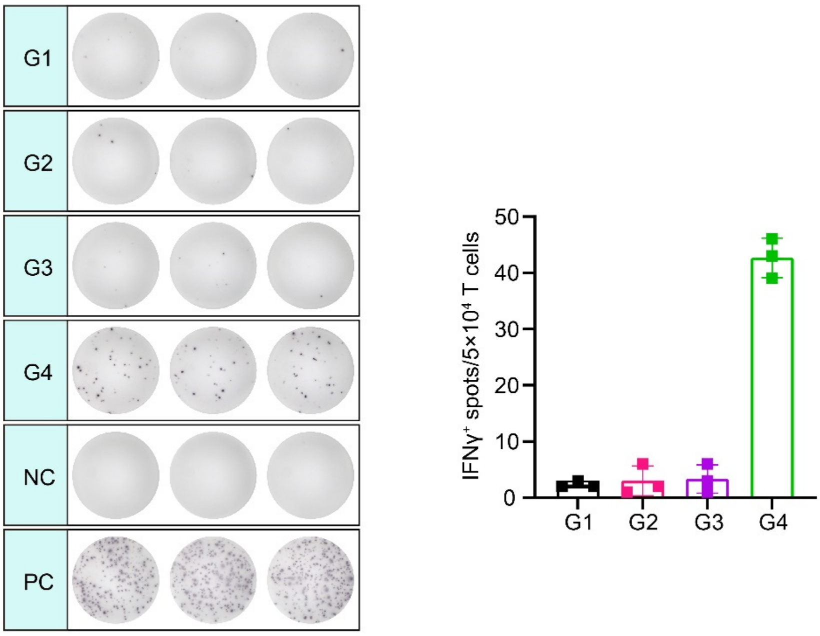

Appendix Fig. S9 ELISpot assay validated the neoantigen responsiveness of the TILs obtained from mice treated with the integrative immunotherapy.

C57BL/6 mice were immunized with Vac-Ctrl or Vac-NF 14 days before the inoculation of B16F10 cells (3×10^6 cells per mouse). The established tumors were intratumorally treated with therapeutic adenoviruses (Adv-Ctrl or Adv-NFH) at 10^9 VP for 5 times. The spleens and treated tumors were obtained on day 20 post the inoculation of B16F10 cells for further analysis. Following the cell isolation, TILs (5×10^4 cells) were subjected to ELISpot assay by co-culturing with DCs pulsed with Ptpn2 peptides (4 μg per 10^4 cells, 24 h) for 24 h. As described in the manuscript, experiments were performed according to the manufacturer's instructions (Mabtech, 3321-4APT-2). NC: negative control in which DCs were treated with PBS instead of peptides. PC: positive control, where TILs were stimulated with anti-CD3ε (2.5 μg/ml) and anti-CD28 (1 μg/ml) (n=3). Data are shown as mean±SD.

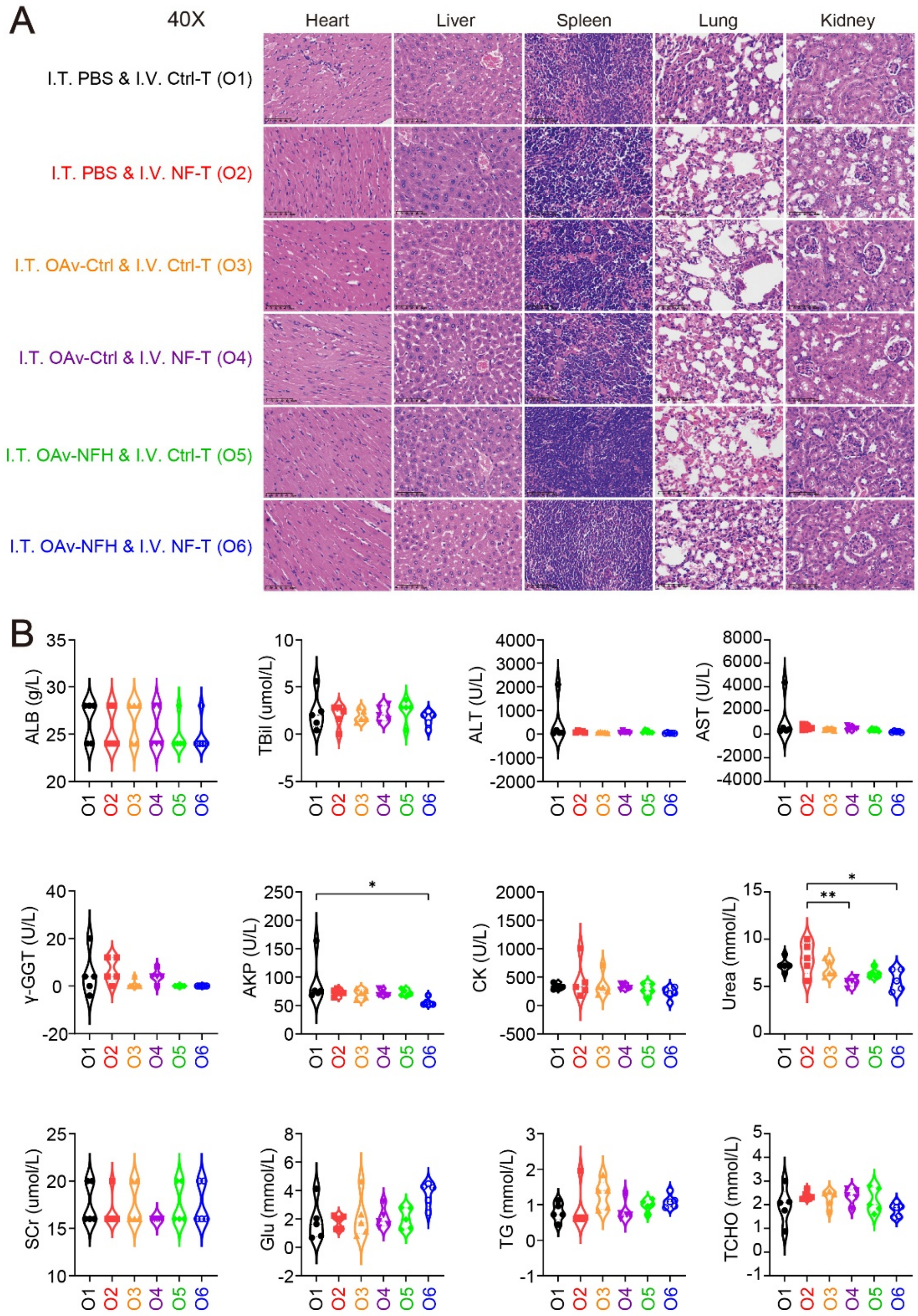

Appendix Fig. S10 Safety of the integrative therapy in BALB/c nude mice.

(**A**) HE staining of mouse organs after the integrative therapy with oncolytic adenoviruses. Scale bar: 50 μm. (**B**) Serum biochemical analysis after the integrative therapy with oncolytic adenoviruses (n=5). Except for AKP and Urea, where significant differences were symbolized by asterisks in the figures, no noticeable differences were observed in other biochemistry indices. Statistical analysis was performed using one-way ANOVA. Data are shown as violin plot for 5 biological replicates.

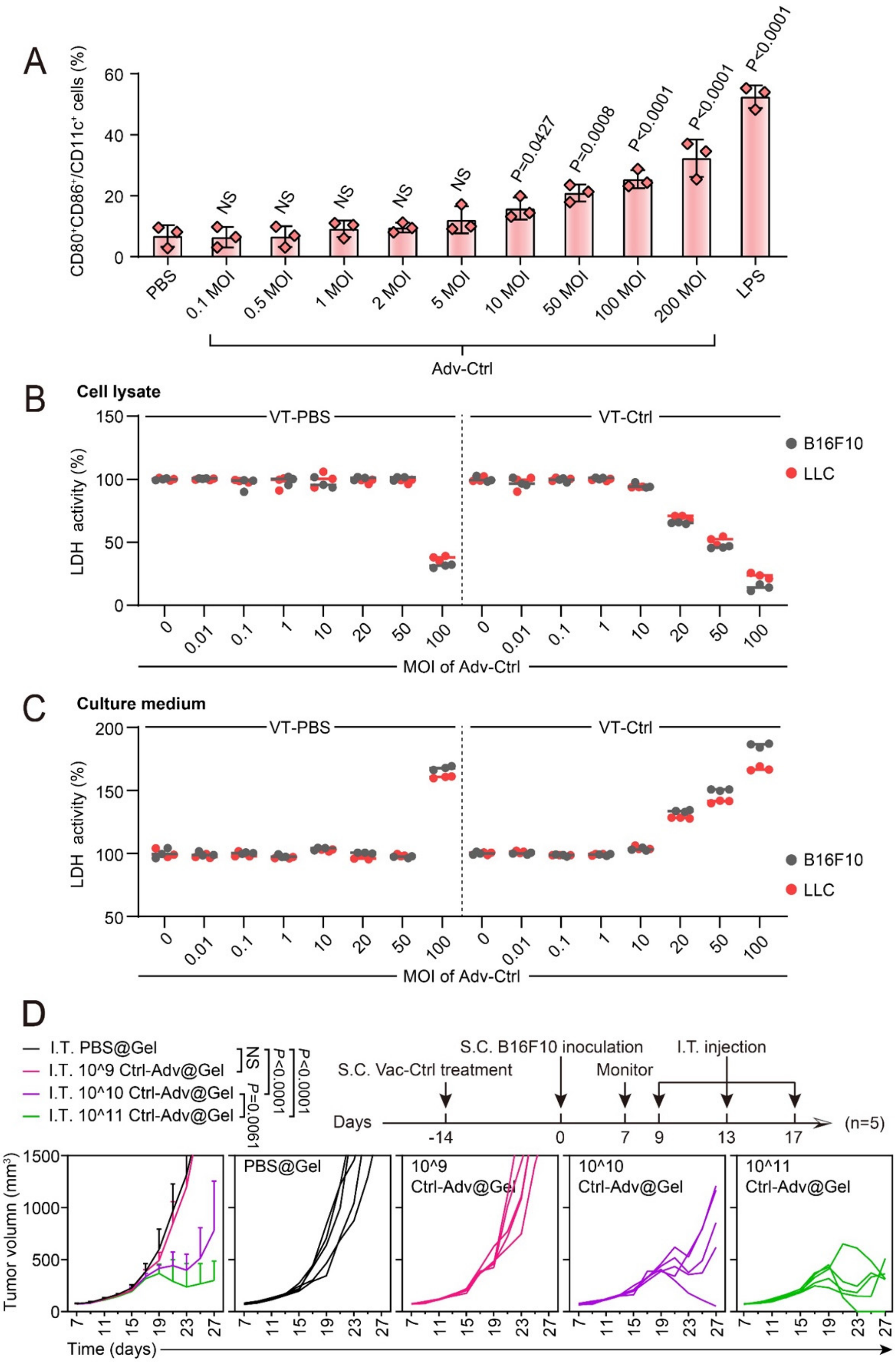

Appendix Fig. S11 Viral backbone induced cellular immunity.

(**A**) Quantification of Adv-Ctrl induced DC maturation. DCs obtained from C57BL/6 mice were treated with PBS, LPS (1 µg/ml), or the indicated MOI of Adv-Ctrl in a 24-

well plate for 72 h before flow cytometry analysis (n=3). (**B** and **C**) LDH assay in lysate of tumor cells (B) and culture medium (C) demonstrated the cytotoxicity of Adv-Ctrl-primed T cells (VT-Ctrl) against Adv-Ctrl-infected tumor cells (n=3). After the infection by indicated MOI of Adv-Ctrl for 48 h, B16F10 and LLC cells were co-cultured with VT-Ctrl at a ratio of 1:3 for additional 48 h. (**D**) Overall and individual tumor growth curves demonstrated the relationship between viral dose and the intensity of virus-specific cellular immunity (n=5 mice per group). An integrative treatment was performed in a subcutaneous B16F10 tumor model with S.C. vaccination (Vac-Ctrl, 2×10^9 VP) and I.T. virotherapy (PBS or Adv-Ctrl). For I.T. injection, Adv-Ctrl (10^9, 10^{10}, or 10^{11} VP) was prepared in 100 μl injectable silk hydrogel. Statistical analysis was performed using two-way ANOVA (D). Data are shown as mean±SD for 3 biological replicates (A, B and C).
