# Supplementary material for: Generalization of neoantigen-based tumor vaccine by delivering peptide-MHC complex via oncolytic virus
Source: EMBO Mol Med. 2025 Apr 7;17(5):1118–52. doi: 10.1038/s44321-025-00225-3 (PMC12081622; doi:10.1038/s44321-025-00225-3)
Supplement: Supplementary file 10 — Expanded View Figures [file 44321_2025_225_MOESM10_ESM.pdf]

Expanded View Figures

Figure EV1. Expression of B2M, H-2Kb and the neoantigens in murine and human cell lines.

(**A**) With indicated primers, the relative expression of B2M and H-2Kb was quantitatively analyzed in HEK293T, HepG2, SK-Hep-1, SMMC-7721, Hepa1-6, B16F10, MC38, and LLC cells ($n = 3$). (**B**) LLC and B16F10 cells were treated with PBS, Adv-Ctrl (1 MOI), Adv-NFH (1 MOI), or Adv-NGSH (1 MOI) for 24 h. The cells were collected and stained by a H-2Kb antibody (Biolegend, 116505, clone AF6-88.5, 5 μg/ml) for 30 min before subjecting to flow cytometry analysis. (**C**) Sanger sequencing results demonstrated the existence of the neoantigens in indicated cell lines. Data are shown as mean ± SD for 3 biological replicates.

 © The Author(s)

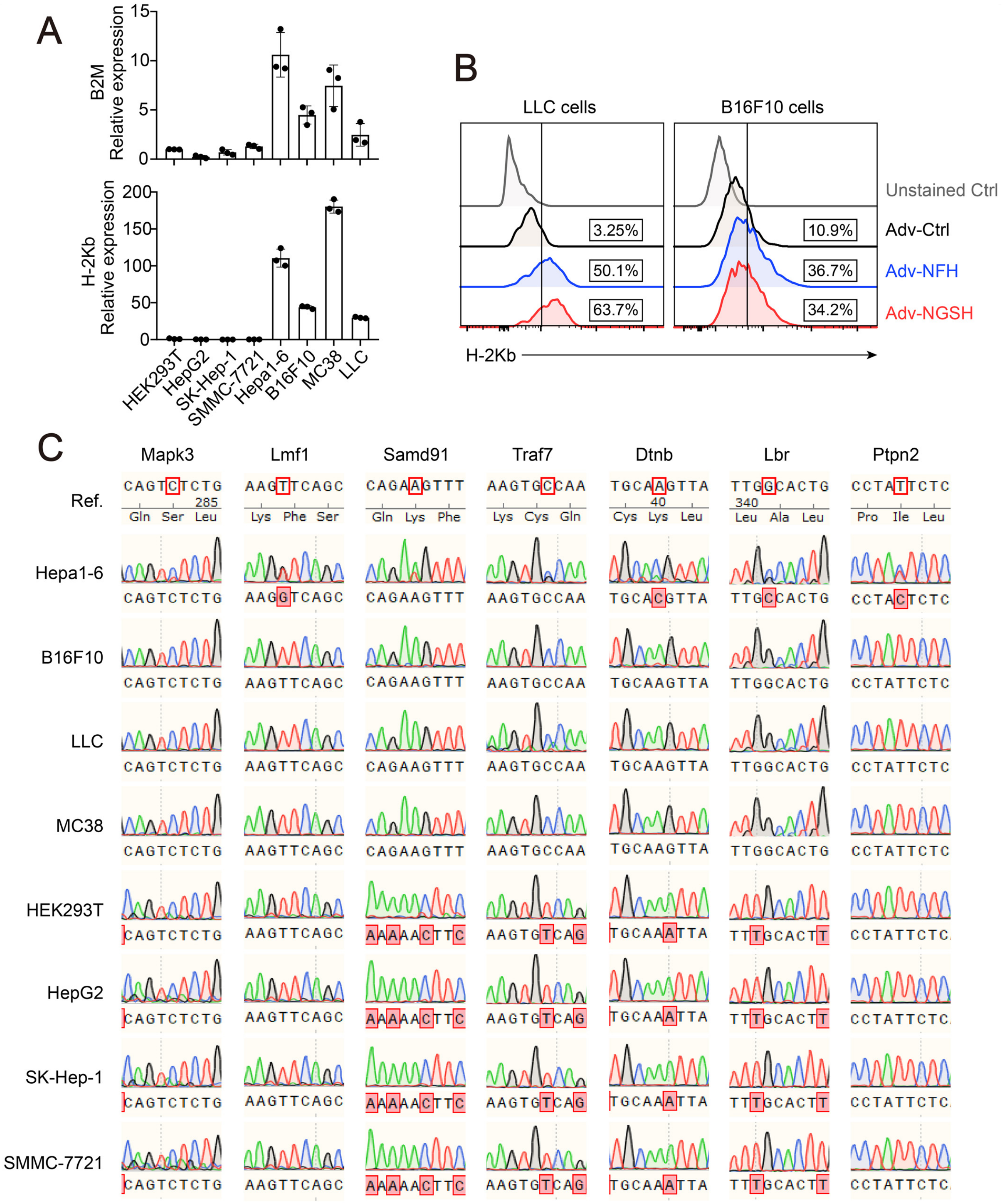

A
B2M Relative expression
H-2Kb Relative expression
HEK293T
HepG2
SK-Hep-1
SMMC-7721
Hepa1-6
B16F10
MC38
LLC
B
LLC cells
B16F10 cells
Unstained Ctrl
Adv-Ctrl
Adv-NFH
Adv-NGSH
3.25%
50.1%
63.7%
10.9%
36.7%
34.2%
H-2Kb
C
Mapk3
Lmf1
Samd91
Traf7
Dtnb
Lbr
Ptpn2
Ref.
CAGTCTCTG
AAGTTCAGC
CAGAAGTTT
AAGTGCCAA
TGCAAGTTA
TTGGCACTG
CCTATTCTC
Gln Ser Leu
Lys Phe Ser
Gln Lys Phe
Lys Cys Gln
Cys Lys Leu
Leu Ala Leu
Pro Ile Leu
Hepa1-6
B16F10
LLC
MC38
HEK293T
HepG2
SK-Hep-1
SMMC-7721

© The Author(s)

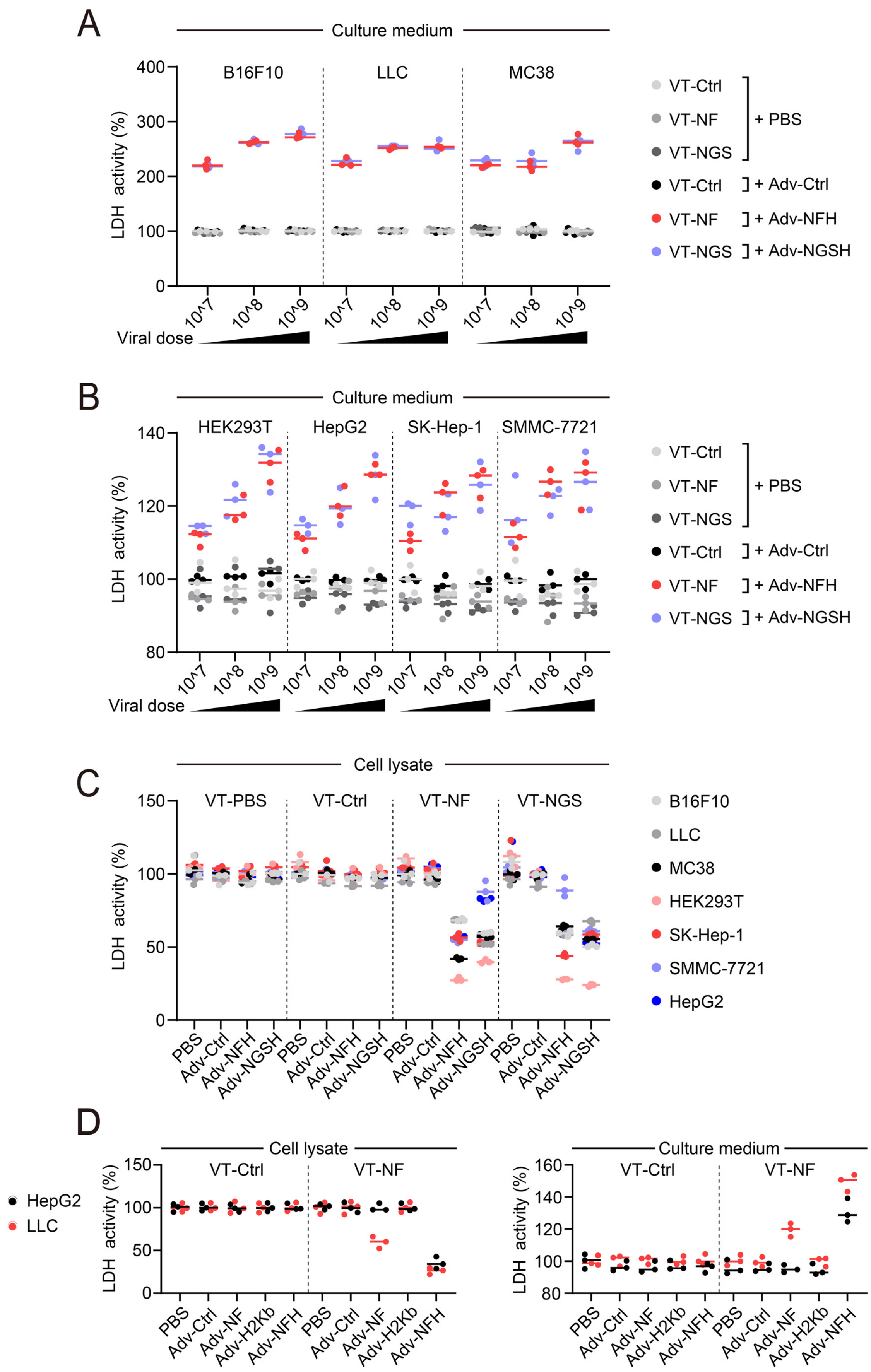
A
Culture medium
B16F10
LLC
MC38
LDH activity (%)
Viral dose
VT-Ctrl
VT-NF
VT-NGS
+ PBS
VT-Ctrl + Adv-Ctrl
VT-NF + Adv-NFH
VT-NGS + Adv-NGSH
B
Culture medium
HEK293T
HepG2
SK-Hep-1
SMMC-7721
LDH activity (%)
Viral dose
C
Cell lysate
VT-PBS
VT-Ctrl
VT-NF
VT-NGS
LDH activity (%)
PBS
Adv-Ctrl
Adv-NFH
Adv-NGSH
B16F10
LLC
MC38
HEK293T
SK-Hep-1
SMMC-7721
HepG2
D
HepG2
LLC
Cell lysate
Culture medium
VT-Ctrl
VT-NF
LDH activity (%)
PBS
Adv-Ctrl
Adv-NF
Adv-H2Kb
Adv-NFH

 © The Author(s)

Figure EV2. Delivery of neoantigen peptide-MHC complexes redirected the cytotoxicity of neoantigen vaccine-primed T cells.

(**A**) LDH assay demonstrated the dose-dependent cytotoxicity of adenovirus-primed T cells in murine tumor cells that infected with Adv-NFH or Adv-NGSH. Samples were the culture medium of the murine cells assessed in Fig. 2E ($n = 3$). (**B**) Dose-dependent cytotoxicity of adenovirus-primed T cells in human tumor cells infected by Adv-NFH or Adv-NGSH. Samples were the culture medium of the human cells used in Fig. 2E ($n = 3$). (**C**) LDH assay in remnant tumor cells showed the specificity of the redirected cytotoxicity ($n = 3$). The assay was performed by following the experimental protocol in Fig. 2E. Mice were vaccinated with 2×10^9 VP of adenoviruses for 14 d before harvesting the SMNCs to prepare related T cells (VT-PBS, VT-Ctrl, VT-NF, and VT-NGS). Tumor cells, including B16F10, LLC, MC38, HEK293T, SK-Hep-1, SMMC-7721, and HepG2, were pre-treated with 1 MOI of indicated viruses and co-cultured with the prepared T cells at an E:T ratio of 1:3 for 48 h. (**D**) LDH assay demonstrated the functionality of MHC-I overexpression. After 48 h infection with 1 MOI of indicated viruses, HepG2 and LLC cells were co-cultured with VT-Ctrl or VT-NF at a ratio of 1:3 for 48 h before assessing the cell viability ($n = 3$). Data are shown as mean ± SD for 3 biological replicates.

© The Author(s)

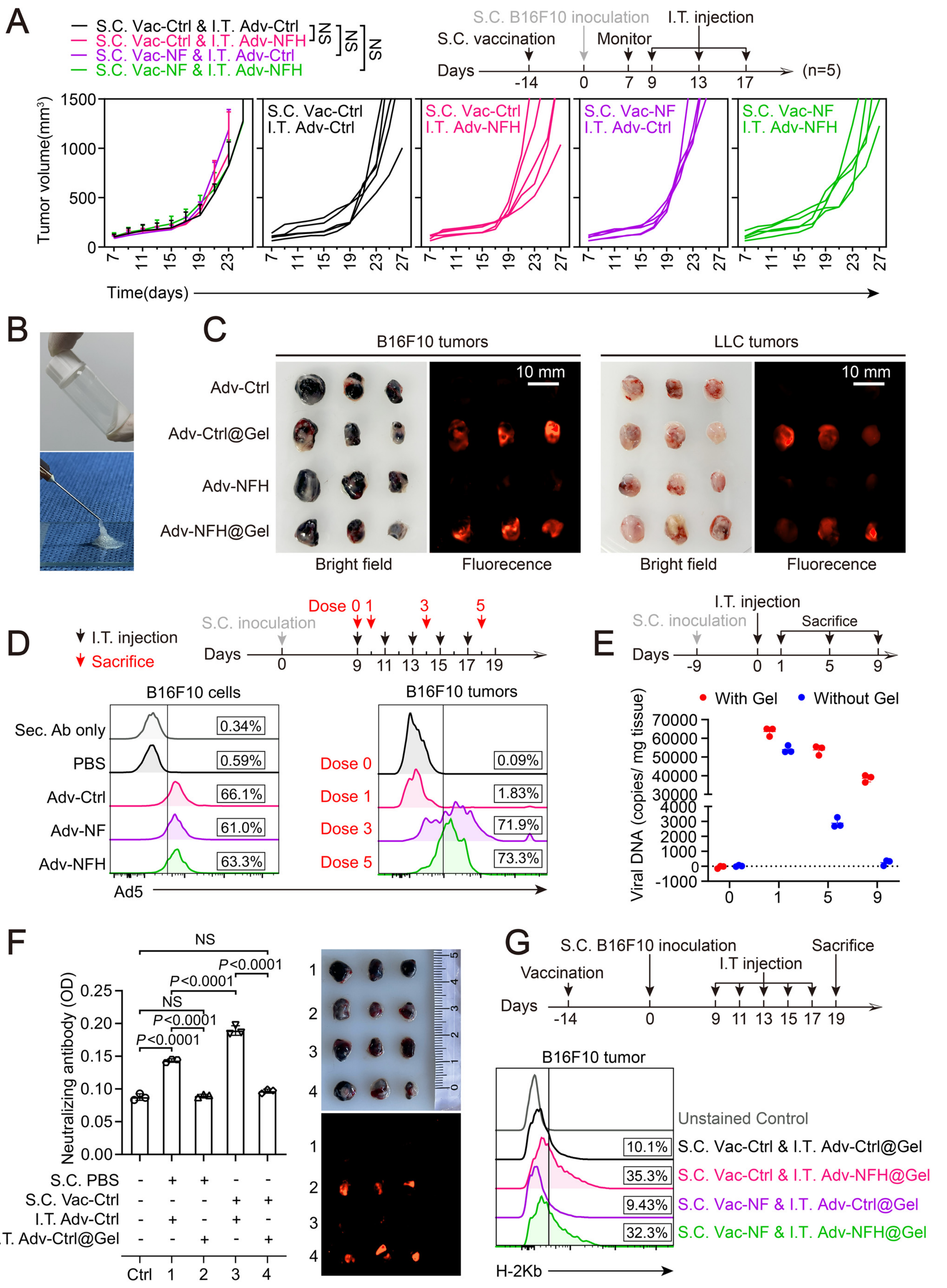
A
S.C. Vac-Ctrl & I.T. Adv-Ctrl
S.C. Vac-Ctrl & I.T. Adv-NFH
S.C. Vac-NF & I.T. Adv-Ctrl
S.C. Vac-NF & I.T. Adv-NFH
NS
NS
NS
S.C. B16F10 inoculation
I.T. injection
S.C. vaccination
Monitor
Days
-14 0 7 9 13 17
(n=5)
Tumor volume(mm³)
Time(days)
S.C. Vac-Ctrl I.T. Adv-Ctrl
S.C. Vac-Ctrl I.T. Adv-NFH
S.C. Vac-NF I.T. Adv-Ctrl
S.C. Vac-NF I.T. Adv-NFH
B
C
B16F10 tumors
LLC tumors
10 mm
Adv-Ctrl
Adv-Ctrl@Gel
Adv-NFH
Adv-NFH@Gel
Bright field
Fluorecence
D
I.T. injection
Sacrifice
S.C. inoculation
Dose 0 1 3 5
Days
0 9 11 13 15 17 19
B16F10 cells
B16F10 tumors
Sec. Ab only 0.34%
PBS 0.59%
Adv-Ctrl 66.1%
Adv-NF 61.0%
Adv-NFH 63.3%
Dose 0 0.09%
Dose 1 1.83%
Dose 3 71.9%
Dose 5 73.3%
Ad5
E
I.T. injection
S.C. inoculation
Sacrifice
Days
-9 0 1 5 9
With Gel
Without Gel
Viral DNA (copies/ mg tissue)
F
Neutralizing antibody (OD)
NS
P<0.0001
P<0.0001
NS
P<0.0001
P<0.0001
S.C. PBS
S.C. Vac-Ctrl
I.T. Adv-Ctrl
I.T. Adv-Ctrl@Gel
Ctrl 1 2 3 4
G
S.C. B16F10 inoculation
Sacrifice
Vaccination
I.T injection
Days
-14 0 9 11 13 15 17 19
B16F10 tumor
Unstained Control
10.1% S.C. Vac-Ctrl & I.T. Adv-Ctrl@Gel
35.3% S.C. Vac-Ctrl & I.T. Adv-NFH@Gel
9.43% S.C. Vac-NF & I.T. Adv-Ctrl@Gel
32.3% S.C. Vac-NF & I.T. Adv-NFH@Gel
H-2Kb

© The Author(s)

Figure EV3. Improving the efficacy of virotherapy with hydrogel encapsulation.

(**A**) Overall and individual tumor growth curves demonstrated an integrative therapy of subcutaneous B16F10 tumor model with S.C. vaccination (Vac-Ctrl or Vac-NF, 2×10^9 VP) and I.T. virotherapy (Adv-Ctrl or Adv-NFH, 10^9 VP) ($n = 5$). (**B**) Representative images demonstrated the successful preparation of injectable silk hydrogel. (**C**) Hydrogel encapsulation potentiated the intratumoral infection of adenoviruses ($n = 3$). (**D**) Quantification of intratumoral infection rate of hydrogel encapsulated Adv-Ctrl at early and late time point with an anti-adenovirus antibody (Merck, AB1056, 1:500) and Alexa FluorTM 488 secondary antibody (Thermo Fisher, A-11055, 2 μg/ml). Before the analysis, the functionality of the primary antibody was validated by staining B16F10 cells that have been infected with adenoviruses (Adv-Ctrl, Adv-NF, or Adv-NFH) in vitro at a MOI of 1 for 24 h (left panel). (**E**) qPCR assay quantified the viral DNA levels in the tumor tissues. Adv-Ctrl was prepared either in hydrogel (With Gel) or PBS (Without Gel) and injected into the tumors. Tumor tissues were subsequently collected at various time points for analysis. (**F**) Adenovirus neutralizing antibody assay and fluorescent imaging showed the protection of adenoviral vectors from host immunity by hydrogel encapsulation ($n = 3$). *P* value: Ctrl vs. 1, $P = 3.380 \times 10^{-7}$; Ctrl vs. 2, $P = 0.982$; Ctrl vs. 4, $P = 0.199$; 1 vs. 2, $P = 4.752 \times 10^{-7}$; 1 vs. 3, $P = 2.034 \times 10^{-6}$; 3 vs. 4, $P = 1.457 \times 10^{-9}$. (**G**) MHC status of B16F10-established tumors following the intratumoral injection of hydrogel encapsulated adenoviruses. Statistical analysis was performed using one-way ANOVA (**F**) and two-way ANOVA (**A**). Data are shown as mean ± SD for 3 biological replicates.

© The Author(s)

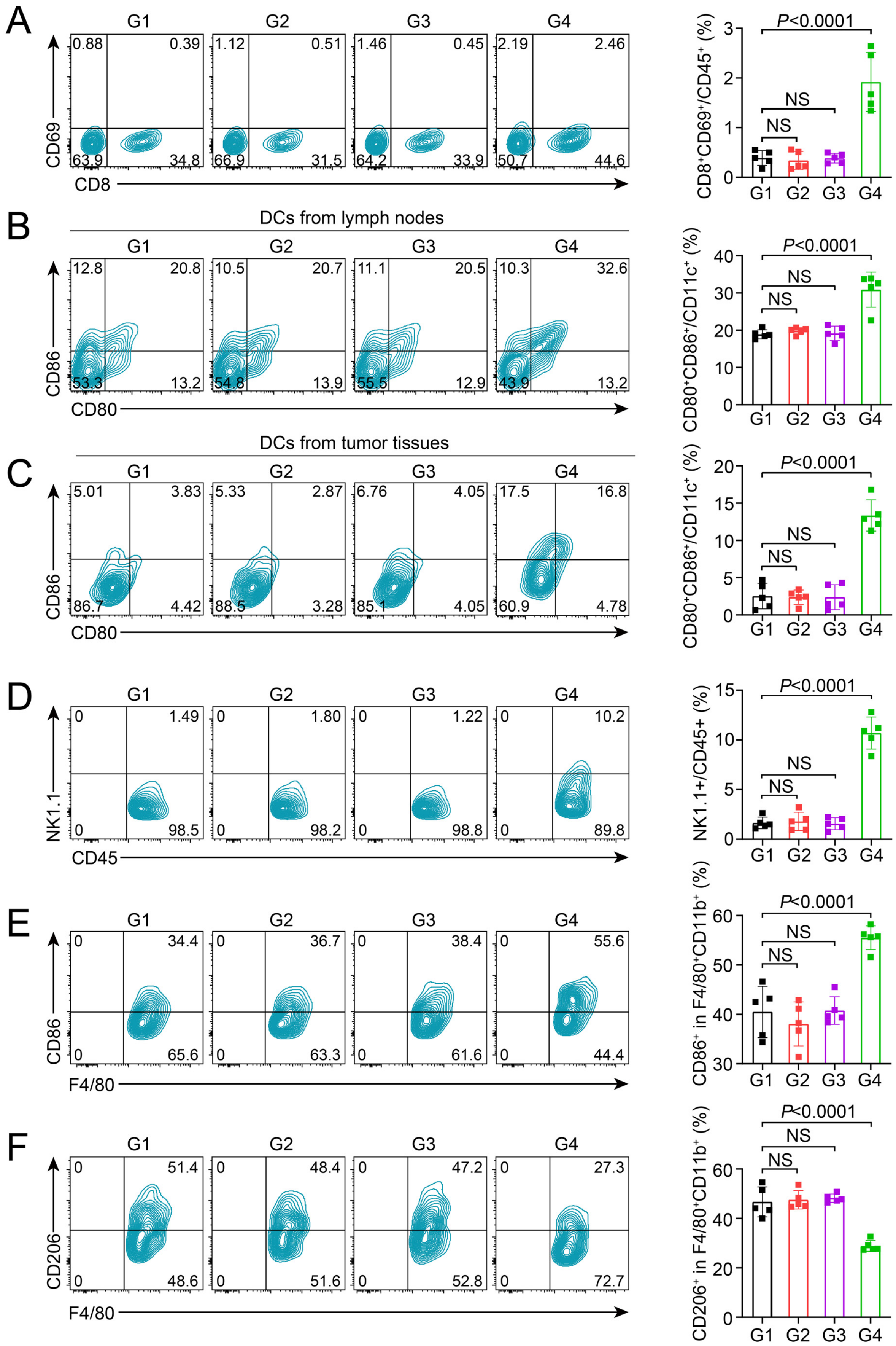
A
G1
G2
G3
G4
CD69
CD8
CD8+CD69+/CD45+ (%)
P<0.0001
NS
NS
B
DCs from lymph nodes
CD86
CD80
CD80+CD86+/CD11c+ (%)
C
DCs from tumor tissues
D
NK1.1
CD45
NK1.1+/CD45+ (%)
E
CD86
F4/80
CD86+ in F4/80+CD11b+ (%)
F
CD206
CD206+ in F4/80+CD11b+ (%)

 © The Author(s)

Figure EV4. Integrative therapy remodeled the tumor microenvironment.

Results of flow cytometry analysis showed the composition of $CD69^{+}CD8^{+}$ T cells in tumors (**A**), $CD80^{+}CD86^{+}$ DCs in lymph nodes (**B**), $CD80^{+}CD86^{+}$ DCs (**C**), $NK1.1^{+}$ NK cells (**D**), $CD86^{+}$ macrophages (**E**) and $CD206^{+}$ macrophages (**F**) in tumors ($n = 5$). PE-Cyanine7-conjugated anti-NK1.1 (eBioscience, 25-5941-82, 2.5 μg/ml), FITC-conjugated anti-F4/80 (BioLegend, 123107, 2.5 μg/ml), APC-conjugated anti-CD11b (eBioscience, 17-0112-82, 1.25 μg/ml), and PE-conjugated anti-CD206 (eBioscience, 12-2061-82, 1.25 μg/ml) antibodies were used for analysis. P value (**A**): G2, $P = 0.995$; G3, $P = 0.999997$; G4, $P = 6.875 \times 10^{-6}$. P value (**B**): G2, $P = 0.946$; G3, $P = 0.999$; G4, $P = 1.359 \times 10^{-5}$. P value (**C**): G2, $P = 0.999$; G3, $P = 0.999$; G4, $P = 1.063 \times 10^{-7}$. P value (**D**): G2, $P = 0.996$; G3, $P = 0.999$; G4, $P = 1.202 \times 10^{-9}$. P value (**E**): G2, $P = 0.755$; G3, $P = 0.9996$; G4, $P = 8.186 \times 10^{-5}$. P value (**F**): G2, $P = 0.987$; G3, $P = 0.924$; G4, $P = 8.309 \times 10^{-6}$. Statistical analysis was performed using one-way ANOVA (**A–F**). Data are shown as mean ± SD for 3 biological replicates.

© The Author(s)

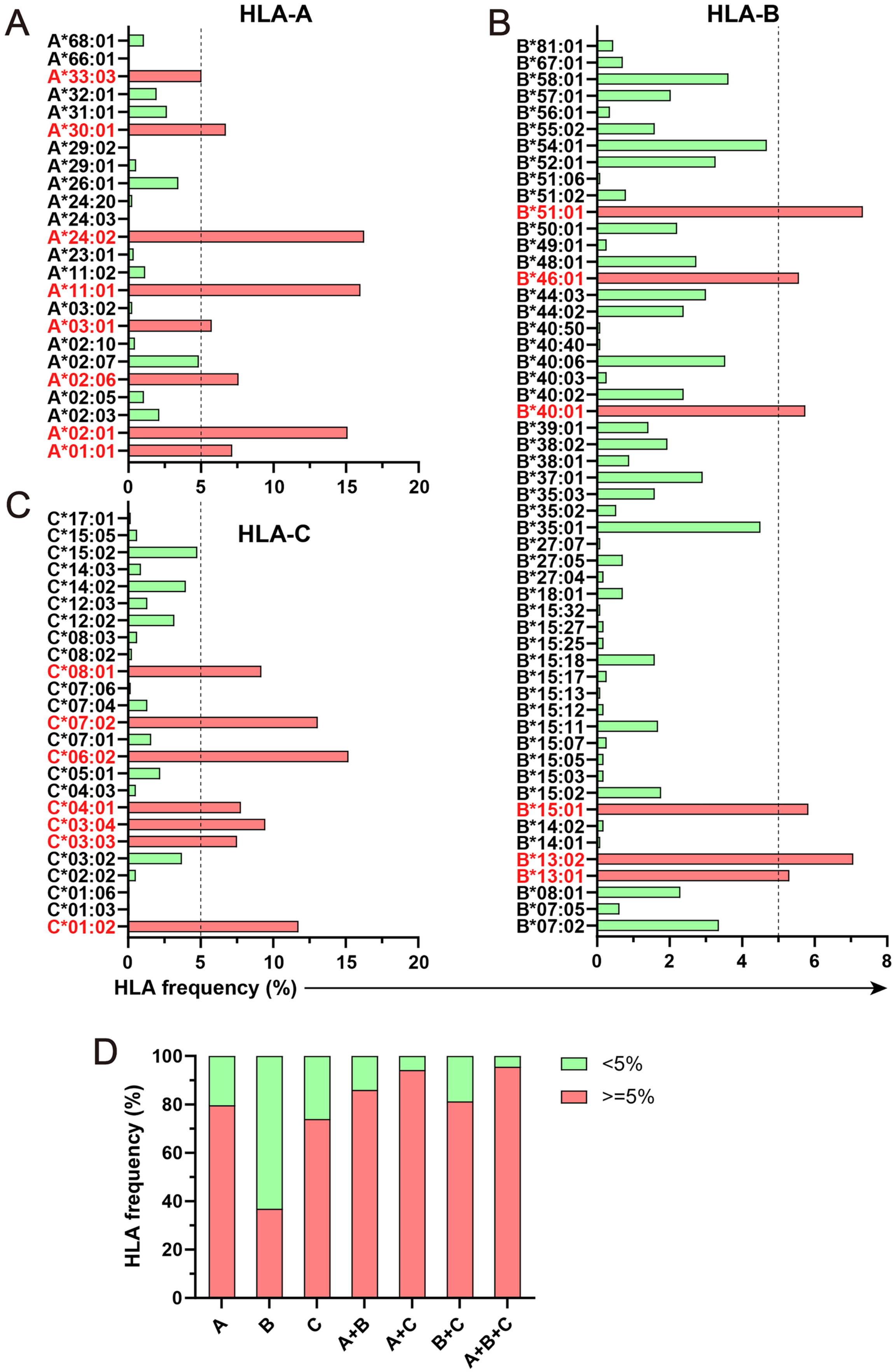
A
HLA-A
A*68:01
A*66:01
A*33:03
A*32:01
A*31:01
A*30:01
A*29:02
A*29:01
A*26:01
A*24:20
A*24:03
A*24:02
A*23:01
A*11:02
A*11:01
A*03:02
A*03:01
A*02:10
A*02:07
A*02:06
A*02:05
A*02:03
A*02:01
A*01:01
0 5 10 15 20
B
HLA-B
B*81:01
B*67:01
B*58:01
B*57:01
B*56:01
B*55:02
B*54:01
B*52:01
B*51:06
B*51:02
B*51:01
B*50:01
B*49:01
B*48:01
B*46:01
B*44:03
B*44:02
B*40:50
B*40:40
B*40:06
B*40:03
B*40:02
B*40:01
B*39:01
B*38:02
B*38:01
B*37:01
B*35:03
B*35:02
B*35:01
B*27:07
B*27:05
B*27:04
B*18:01
B*15:32
B*15:27
B*15:25
B*15:18
B*15:17
B*15:13
B*15:12
B*15:11
B*15:07
B*15:05
B*15:03
B*15:02
B*15:01
B*14:02
B*14:01
B*13:02
B*13:01
B*08:01
B*07:05
B*07:02
0 2 4 6 8
C
HLA-C
C*17:01
C*15:05
C*15:02
C*14:03
C*14:02
C*12:03
C*12:02
C*08:03
C*08:02
C*08:01
C*07:06
C*07:04
C*07:02
C*07:01
C*06:02
C*05:01
C*04:03
C*04:01
C*03:04
C*03:03
C*03:02
C*02:02
C*01:06
C*01:03
C*01:02
0 5 10 15 20
HLA frequency (%)
D
HLA frequency (%)
0 20 40 60 80 100
A B C A+B A+C B+C A+B+C
<5%
>=5%

 © The Author(s)

Figure EV5. Coverage of HLA molecules indicates the applicability of the integrative therapy in human.

Allele frequencies of HLA-A (**A**), HLA-B (**B**), and HLA-C (**C**) in the 741 individuals from Han population residing in Shanxi Province of China. The data was downloaded from PGG.MHC website: https://pog.fudan.edu.cn/pggmhc/#/population, and all the alleles were plotted. The 5% cut-off value was indicated by the dotted line. Alleles with frequencies <5% and >=5% were, respectively, indicated by green and red columns. The coverage of alleles with frequencies >=5% was calculated and demonstrated as cumulative histogram (**D**), in which HLA-A, HLA-B, and HLA-C were, respectively, abbreviated as A, B, and C. The cumulative results (>=5%) were as follows: A (79.59%), B (36.84%), C (73.94%), A + B (85.94%), A + C (94.17%), B + C (81.27%), A + B + C (95.58%).

© The Author(s)
